# Does performance on United States national board of medical examiners reflect student clinical experiences in United Arab Emirates?

**DOI:** 10.15694/mep.2019.000004.2

**Published:** 2019-04-01

**Authors:** Omran Bakoush, Ali Al Dhanhani, Saif Alshamsi, Janet Grant, John Norcini

**Affiliations:** 1 United Arab Emirates University; 2 Centre for Medical Education in Context (CenMEDIC) and Department of Education in Medicine; 3 Centre for Medical Education in Context (CenMEDIC) and Department of Education in Medicine; 4 Foundation for Advancement and International Medical Education and Research

**Keywords:** Clinical teaching, Learning environment, Internal Medicine, National Board of Medical Examiners, Student performance, UAE, Undergraduate education

## Abstract

This article was migrated. The article was marked as recommended.

**Background:** A number of medical schools around the world use the United States National Board of Medical Examiners Subject Examinations as a clerkship assessment of student performance, yet these exams were blueprinted against the United States national core clerkship curriculum which might not be the same as the local curricula to which they are applied in other parts of the world. In this study, we investigated the correlations between the internal medicine clinical experiences at United Arab Emirates University with student performance on the National Board of Medical Examiners subject of internal medicine (NBME).

**Methods:** One hundred and seven junior clerkship students out of 145 (74%) who finished their Internal Medicine clerkship during academic years 2014-2015 and 2015-2016 participated in this study. The students’ clinical experiences were measured by the clinical learning evaluation questionnaire (CLEQ) and by the logged number of meaningful patient contacts during their internal medicine clerkship.

**Results:** Linear regression analysis showed no significant association between performance on the subject test and student clinical experiences measured by the CLEQ or the number of logged patients. NBME scores were weakly correlated with OSCEs scores (ɸ 0.20).

**Conclusions:** The study findings raised uncertainties about the suitability of using NBME in the clerkship assessment program in the United Arab Emirates.

## Introduction

Undergraduate medical curricula strive to produce future doctors equipped with clinical knowledge and skills sufficient to practice and improve patient care. Medical schools use standardized examinations to measure student clinical competency as one check on the curriculum’s effectiveness. The National Board of Medical Examiners Subject Exams (NBMESE) is a highly regarded tool used in the assessment of the clinical knowledge that students acquired during clerkship ( Bass *et al.*, 1997; Elnicki, Lescisin and Case, 2002). Furthermore many reports in the United States (US) highlight the relationship of NBME scores with the quality of clinical teaching and student clinical experiences ( Nahum, 2004; Griffith *et al.*, 2009; Ramchandani, 2011; Dong *et al.*, 2014; Myers *et al.*, 2014; Ouyang, Cuddy and Swanson, 2015; Casey *et al.*, 2016).

Because of NBMESE’s high reliability, benchmarking function, and practicality of administration, many medical schools outside the US, including United Arab Emirates University (UAEU), use the NBME Subject Exams for clerkship assessment of student academic achievement ( Colthart *et al.*, 2008). However, uncertainties exist as to how well the NBMESEs’ scores correlate with the clerkship clinical experiences of medical students in schools outside the US ( Tekian and Boulet, 2015). The differences in clinical learning environment, the patients’ population, and health care practices might contribute to significant variations in clinical teaching and student clinical experiences between countries and even regions within the same country. These differences might alter the utility of the NBMESE as a valid measure of student clinical knowledge acquired during the clerkship outside the US ( Griffith *et al.*, 2009). Interestingly, even some Canadian medical schools report discrepancies between outcomes of NBMESEs and in-house Canadian written exams ( Hermanson *et al.*, 2004; Veale *et al.*, 2011).

The aim of this study was to investigate the relationship between UAEU students’ clinical experiences and their performance on the NBMESE-internal medicine exam to get better understanding of the utility of NBME scores in assessing student clinical knowledge gained from clerkship teaching, as well as the relationship between those scores and student clinical experiences.

Given this background, the objective of this study is to explore the

• Impact of UAEU clinical experiences on the scores of the NBME internal medicine subject examinations;
• Relationship between NBME scores and scores on the in-house objective structural clinical exams (OSCEs);
• Impact of pre-clerkship academic achievement on outcomes of NBME internal medicine subject examinations.

## Methods

The College of Medicine and Health Sciences, United Arab Emirates University (UAEU) is the first medical school to be established in UAE and in recent years it matriculates between 70 and 100 students annually. At the time of this study, UAEU offered a traditional six-year curriculum: two years of pre-medical sciences followed by a two-year pre-clinical program and capped by a two-year clinical program where the core clinical specialties were taught in clerkships.

The internal medicine clerkship program occurs in teaching hospitals affiliated with UAE University. The clinical teaching activities over and above apprenticeship include bedside teaching, hospital ward rounds, night duties, small group conferences and case discussions. The clerkship program assesses students’ clinical competencies by exams designed in-house, including an objective structured clinical exam (OSCE). The subject examinations provided by NBME are used for assessment of clerkship cognitive learning outcomes. The NBMESEs assess the student’s understanding and ability to apply clinical knowledge in relation to the physician’s tasks of solving patient problems and promoting health ( NBME, 2016). The NBMESE-internal medicine is an obligatory end of clerkship exam for junior clerkships at UAEU since 2009. The NBME reported that the norm group mean scores for internal medicine were 73.3 (±9) for the academic year 2014-2015 and 74.7 (±8.9) for the academic year 2015-2016.

The study sample was limited to junior clerkship medical students’ in the academic years of 2014‒2015 and 2015-2016. A sample size of 106 respondents would yield results within +/- 5% confidence intervals at 95% confidence level.

Data collected for each student included demographics, post-clerkship exam scores on NBME and OSCEs, and the pre-clerkship scores of the final end of pre-clinical-years comprehensive exit written exams. The internal medicine junior clerkship coordinators provided the scores for NBME and OSCEs for each student who rotated on Internal Medicine junior clerkship during the academic years 2014-2015 and 2015-2016. The pre-clerkship scores were provided by the examination office of the College of Medicine and Health Science. We studied the influence of prior academic achievement by using the pre-clerkship scores as a control variable. High achievers and low achievers were defined as students with a pre-clerkship score above or below the mean score of the study cohort.

### Student Clinical experiences

The Clinical Learning Evaluation Questionnaire (CLEQ) was used to measure student clinical teaching experiences (Appendix 1). The CLEQ measures the student perception of the quality of undergraduate clinical teaching in a clinical placement. The students score the number and quality of the clinical cases they see, their involvement in the patient care, and the quality of clinical supervision and organisation of the patient-doctor encounter. The CLEQ was validated to measure the effectiveness of undergraduate clinical teaching in Saudi medical schools and thus was deemed to be sensitive enough to measure the effectiveness of undergraduate clerkship clinical teaching in the UAEU local context ( Al-Haqwi, Kuntze and vander-Molen, 2014).

In a selected response format, the students reported the number of meaningful patient encounters they had. A meaningful patient encounter is defined as involvement of the student in interviewing the patient or performing the physical examination and not where a patient is described or presented in the student’s presence. The students were asked to rank on a Likert scale the importance of teaching methods; textbook and online resources, self-practice (solving MCQs questions, practicing clinical skills), faculty teaching sessions (morning report, tutorial, etc.), and clinical experiences (patient workups, bedside teaching, etc.) in preparation for the post clerkship examinations of NBMESE and OSCEs.

### Administration of the questionnaire

To determine local contextual relevance, the questionnaire was piloted on 11 medical clerkship students. It was validated for the time required to complete it, whether the questions were appropriate, clear, concise, and unambiguous, and whether the instructions were easy to understand and meaningful. All the students were able to answer the questionnaire within 10 minutes.

The questionnaire was administered on 27 September 2015 using online survey software, SurveyMonkey ( http://www.surveymonkey.com/). The respondents were instructed that their responses to the questions should be based on their experience during their clinical rotations and not on their general impressions. Completion of the survey signified informed consent. The research protocol was approved by the ethical committee of Al Ain district in UAE (Number: 15-3088). A weekly reminder was sent (a total of five reminders), but no incentives were provided ( Cohen, Manion and Morrison, 2013).

### Statistical analysis

The respondents received identification codes, so their response data and examination scores were entered into analyses without using their names. The mean values and standard deviations (SD) were calculated for the continuous variables of the scores of the NBMESEs, scores of the OSCEs, final clerkship scores, and the final pre-clinical scores. To allow comparisons, the scores of pre-clerkship and end-clerkship exams were converted to Z scores (10 (Z) +70).

The rating of CLEQ items in Likert format produced ordinal data. Therefore, medians and interquartile ranges (IQR) were used to calculate the central measure summary for the scores of the items and the subscales of the CLEQ-measure. A reverse score was counted for the negative statements.

We performed a univariate linear regression analysis to investigate the effect of the pre-selected potential variables on student academic achievement. The outcome of interest was student score on the NBMESE as continuous variable ( Table 1). The independent variables were the scores of the CLEQ subscales, the number of patient contacts, student gender, and the pre-clerkship scores.

Comparisons of continuous variables were made using Student’s t test. R square (R ^2^) values represented the predictive power of the pre-clerkship scores for student performance, and the predictability of NBMESE for OSCE performance. Mann-Whitney and Pearson Chi square tests were used to compare study subgroups, as appropriate. The Statistical Package for the Social Sciences (IBM SPSS; Version 21) was used for all statistical analyses. Test statistics with P values less than 0.05 (two-sided) were considered statistically significant.

## Results/Analysis

One hundred and forty-five medical students were invited to participate in this study. The ages of the 145 invited students ranged from 22 to 27 years, with an average of 23.3 years (SD ± 0.72). Twenty-six of them (17.9%) were men. Out of the 145 students who were invited, 107 students agreed to participate and completed the questionnaire, giving a response rate of 74%. There is no significant difference between responders (n = 107) and non-responders (n = 38) in age, or scores of pre-clerkship or end of Internal Medicine clerkship exams ( Table 1).

**Table 1.** Comparison of responding and non-responding CMHS fifth-year medical students during the academic years 2014 - 2016.

	Responders	Non-Responders	P-value
Gender (n: M/F)	23/84	20/18	0.001
Age	23.22 (0.61)	23.40 (0.76)	0.10, ns
Pre-clerkship scores	82.40 (5.03)	80.91 (5.38)	0.12, ns
NBME score	46.17 (11.42)	44.71 (11.39)	0.50, ns
OSCEs score	81.30 (5.42)	79.48 (6.07)	0.09, ns

Except for gender, values are given as mean (standard deviation).

Overall the respondents rated the internal medicine clerkship to be satisfactory in providing clinical cases for teaching, adequate clinical supervision, and organisation of doctor-patient encounters (IQR 4-5). However, the authenticity of the clinical experiences was rated to be less satisfactory (IQR 3‒5, p = 0.002). The number of patients examined by the students varied widely between individual students. On average, each student clerked a minimum of 33 patients (SD ± 14) during the 8 weeks of the internal medicine junior clerkship.

Table 2 shows the impact of pre-clerkship achievement on the student clinical experience. The students’ perceptions of the qualities of the clerkship learning environment were independent of their prior academic achievements, as there was no significant difference between high and low achievers in their ranking of the CLEQ items or the number of meaningful patient contacts they had during the internal medicine junior clerkship rotation.

**Table 2.** The interquartile ranges of scores of students’ ratings for CLEQ measure, the mean number of contacts with patients, and the mean scores of clerkship exams, of low achievers compared to high achievers.

	Total	Low achievers	High achievers	P-value
Number (males/females)	23/84	15/37	8/47	0.055
**CLEQ subscales**				
Provision of the clinical cases	4-5	4-5	3.5-5	0.97, ns
Authenticity of clinical experience	3-5	3-5	3-4	0.19, ns
Clinical Supervision	4-5	4-5	4-5	0.48, ns
Organization of doctor-patient encounter	4-5	4-5	4-5	0.60, ns
Motivation to learn	4-5	4-5	4-5	0.43, ns
Overall CLEQ scale	4-5	4-5	4-5	0.82, ns
**Log of patient- encounters**				
History taking	33.10 (14.15)	31.40 (13.63)	34.64 (14.56)	0.24, ns
Physical examination	27.89 (12.93)	26.67 (13.05)	29.15 (12.84)	0.38, ns
**Exam performance**				
NBME	46.17 (11.42)	39.50 (7.41)	52.47 (10.98)	**< 0.001**
OSCEs	81.30 (5.42)	79.33 (5.23)	83.18 (4.96)	**< 0.001**
Total clerkship score	82.26 (4.39)	79.83 (3.98)	84.56 (3.42)	**< 0.001**
**Above average performance**				

High achievers and low achievers were defined as students with a pre-clerkship score above or below the mean score of 81.6% for the study cohort.

The scoring scale for CLEQ items 1-5 (1= poor, 2= fair, 3= good, 4= good, 5= excellent).

**NBMESE performance:** The study group overall mean NBMESE score was low compared to the NBME norm group (46±11 *vs* 74±9, p<0.001). Linear regression analysis showed no significant association between NBME performance and clinical experiences as measured by the CLEQ or number of logged patients ( Table 3).

**Table 3.** Univariate linear regression analysis: Predicting the outcome of NBME internal medicine subject exam.

Variables	Beta	SE	t-value	p-value
Provision of the clinical cases	0.171	1.42	0.50	0.62
Authenticity of clinical experience	-1.16	0.96	-1.21	0.23
Clinical Supervision	1.003	1.27	0.79	0.43
Organization of clinical teaching	-0.56	1.89	-0.29	0.77
Motivation to learn	2.04	1.62	1.26	0.21
Number of patient contact	0.011	0.081	0.14	0.89
Female gender	7.91	2.59	3.06	**0.003**
Pre-clerkship achievements	1.63	0.16	10.50	**<0.001**

Beta= Regression Coefficient; SE= standard error; t= t-value for beta.

Female gender (β= 7.9, p = 0.003) and pre-clerkship scores (β = 1.6, p < 0.001) were associated with better student performance on NBMESE. The pre-clerkship grades were a better predictor of the variation in NBMESE scores (51%, R ^2^ = 0.51), than the variation in OSCE scores (20%, R ^2^ = 0.20), or the variations in the local MCQ exams (25%, R ^2^ = 0.25).

Student performance in NBMESE was weakly correlated with their performance in OSCEs (phi correlation coefficient ɸ 0.20), and with their performance in local MCQ (phi correlation coefficient ɸ 0.26). The ability of NBMESE scores to predict OSCE outcomes (11%, R ^2^ = 0.11) and local MCQ outcomes (19%, R ^2^ = 0.19) was limited. A substantial number (41%) of UAEU students who scored below the class mean on the NBMESE were able to score above the class mean in OSCEs, and 39% of the students who scored above the class mean in NBMESE performed below the class mean in OSCEs ( Table 4). The same figures were applied to local MCQ exams ( Table 4).

**Table 4.** Cross tabulation: predictability of NBME performance for OSCEs outcome and in-house-MCQ outcome.

NBME outcome	OSCEs outcome		In-house MCQ outcome		
	Below class mean	Above class mean	Below class mean	Above class mean	
**Below class mean**	34 (58.6%)	24 (41.4%)	35 (60.3%)	23 (39.7%)	58
**Above class mean**	19 (38.8%)	30 (61.2%)	17 (34.7%)	32 (65.3%)	49
	53	54	52	55	107

**Figure 1.**
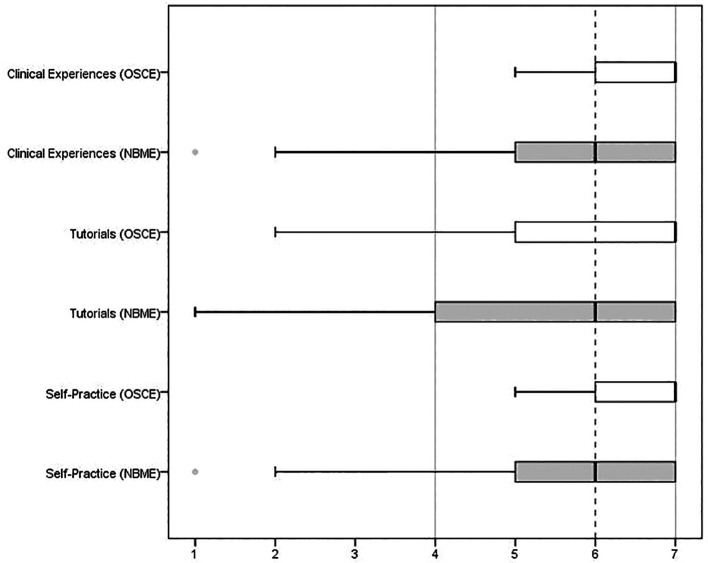
Medians and interquartile ranges of student rating for the importance of clinical teaching methods in preparation for NBME and OSCE.

On a Likert scale, the students ranked their clinical experiences, teaching sessions, and self-practice to be more important in preparation for OSCEs than the NBMESE exam (p = 0.001, Figure 1). They perceived reading textbooks and online resources to be more helpful in preparation for NBMESE exams than the clinical teaching.

1= least important, 7= most important

## Discussion

Our study examined the relationship between UAEU student NBMESE performance and their clinical experiences acquired during internal medicine clerkship. Student clinical experiences were measured empirically using the newly constructed clinical learning environment questionnaire (CLEQ) which was deemed to be the most suitable for the UAE context ( Al-Haqwi, Kuntze and vander-Molen, 2014).The study findings indicated the limited ability of NBMESE to predict student clinical experiences in UAE. The NBMESE scores have a low degree of correlation with UAEU clinical experiences and the UAEU student scored on average 37% lower than the NBME norm cohort, Table 1.

The study finding of the association of the pre-clerkship scores with better performance on NBMESE exams is in line with the recently published report on the correlations of the scores of the NBMESE exams with the scores of the USMLE step-1 pre-clerkship exam in US ( Casey *et al.*, 2016). This association could be related to the similarity of the NBMESE and pre-clerkship exams in assessment of student understanding of the mechanisms of disease. However, the other NBMESE domains related to clinical tasks such diagnosis and management could be less related to the UAEU clinical learning experiences. In their preparation for the NBMESE, the respondents indicated that reading medical textbooks and online resources are of more importance than their clerkship clinical experiences. The NBMESE as a high stake examination for UAEU students could be potentially counterproductive as it might have created considerable stress for students trying to maintain a balance between reading textbooks to pass NBME and seeing more patients which would improve their clinical experience ( Cobb *et al.*, 2013).

A recent study by Holtzman et al (2014) reported a wide variation in the performance of international medical students in the domains of the clinical tasks compared to domains of mechanism of diseases related to the text book knowledge ( Holtzman *et al.*, 2014). Although, the UAEU undergraduate medical curriculum is benchmarked with the US national core medical curriculum, the difference in patient population and sociocultural factors might have contributed to the disparity between the UAEU student NBMESE performance and the US student NBME performance ( Griffith *et al.*, 2009). To enhance student clinical learning, the clerkship exams should be blueprinted to their contextual clinical experiences, the patient population, and the contextual approach for solving patients’ problems ( Lucas, Benedek and Pangaro, 1993; McManus *et al.*, 1998; Cilliers *et al.*, 2010; Rentea *et al.*, 2015; Archer *et al.*, 2016).

The results of this study must be interpreted with caution due to several limitations. The study covered a relatively small sample of students and data were collected only from the junior internal medicine clerkship rotation. It would have been a stronger study if other UAEU clerkships were included and other NBMESEs considered. The student perceptions of clinical experience could be biased by single outstanding events rather than reflecting the quality of the actual clinical teaching experiences ( Woloschuk *et al.*, 2011; Schiekirka and Raupach, 2015). Also the perceived low authenticity of the clinical experiences could have negatively affected student academic performance, as authenticity reflects the degree of student inclusion into the clinical placement climate ( Billett, 2002; Strand *et al.*, 2013).

The reliability of NBMEs is reported to be high, but the reliability coefficient of our in-house OSCEs is not measured ( Hoffman, 1993). Processes that assure the good reliability of our in-house OSCEs include the use of faculty as examiners, standardised scoring rubrics with global rating, an adequate number and length of OSCEs stations, and frequent reuse of volunteer patients from the examination pool ( Pell *et al.*, 2010).

Despite these limitations, its low student performance and low degree of correlation with UAEU clinical experiences might indicate a low degree of utility for use of NBMESEs as a summative assessment tool for internal medicine clerkship in UAE ( Fielding, 2008; Dong *et al.*, 2014; Casey *et al.*, 2016). If these results are confirmed through further study, the scores of the NBMESEs should not be used to determine pass-fail decisions in UAEU and the relative weight of NBMESE scores in the computed final composite clerkship scores should be considered carefully ( Fielding, 2008). Creating a national standardised written clerkship exam blueprinted against UAE clinical learning experiences would have a positive impact on future Emirati doctor clinical experience.

## Conclusion

The ability of NBME scores to reflect on the UAE student clinical experience is modest. A longitudinal cross-clerkship evaluation that includes other core clerkships would be necessary to provide more reliable information on the value of NBMESE to assess the student clinical experiences across multispecialty clinical learning environment in UAE.

## Take Home Messages

• Low correlation between UAEU students’ performance in NMBESE and their clinical experiences.
• Reading text books is perceived to be more important in preparation for NBMESE exams than UAEU clinical teaching.
• NBMESE as summative clerkship exam might adversely influence student study habit and clinical learning environment in UAEU internal medicine clerkship.

## Notes On Contributors

**Dr Omran Bakoush**: Professor & Consultant of Nephrology, Department of Internal Medicine, College of Medicine & Health Science, UAEU, Al Ain, UAE. ORCID https://orcid.org/0000-0003-1447-9836

**Dr Ali Al Dhanhani**: Assistant Professor & Consultant of Rheumatology, Department of Internal Medicine, College of Medicine & Health Science, UAEU, Al Ain, UAE.

**Dr Saif Alshamsi**: Assistant Professor & Consultant of Internal Medicine, Department of Internal Medicine, College of Medicine & Health Science, UAEU, Al Ain, UAE.

**Prof Janet Grant**: Director of the Centre for Medical Education in Context (CenMEDIC) and Emeritus Professor of Education in Medicine at Open University, UK.

**Dr John Norcini**: President & CEO of Foundation for Advancement and International Medical Education and Research, Philadelphia, USA.

## Appendices

### Appendix 1: The study questionnaire

The students asked to rank on scale 1-5 (1= poor, 2= fair, 3= good, 4= good, 5= excellent) the following CLEQ factors:

#### Part I: The clinical learning environment questionnaire (CLEQ)

##### Factor 1: Provision of cases:

• I have seen a sufficient number of clinical cases.
• I have seen a sufficient number of new clinical cases.
• I have seen a good variety of clinical cases.
• I have seen many interesting clinical cases.
• I have seen some cases with positive clinical findings.
• I have seen some unusual/rare clinical cases.

##### Factor II: Authenticity of clinical experiences:

• I have the opportunity to have the first contact experiences with patients.
• I am actively involved in the patient care.
• I have the opportunity to deal with patient as a real doctor.
• I have the opportunity to deal with the patient as a whole and not limited to a certain system or organ.
• I have the opportunity to apply my previous knowledge in patient care.
• I have the opportunity to apply a patient-centred approach.
• I have the opportunity to take responsibility for patient care.
• I have the opportunity to communicate with patients and their families.

##### Factor III: Clinical supervision:

• My supervisors have good communication skills.
• I have been respected by my supervisors.
• The supervisors are committed for teaching.
• The way my supervisors deal with medical students is satisfactory.
• I think supervisors have good teaching skills.
• I have rarely received a good feedback on my clinical performance from my supervisor.
• I think that some supervisors could be considered as role models.

##### Factor IV: Organization of the doctor-patient encounter:

• The objectives of the clinical rotations are clear.
• Students have some input for the organization and development of the clinical rotations.
• I have the opportunity to prepare before the clinical encounter.
• I have the opportunity to reflect and read after the clinical encounter.
• I have the opportunity to discuss clinical cases with my supervisors.
• I have the opportunity to share the clinical cases with other students.
• The number of students in the clinical sessions is appropriate.
• The time spent with my patients is adequate for my clinical learning.
• I have the opportunity to utilize skills lab and simulation for clinical cases.
• I think the assessment of the clinical learning is aligned with objectives.
• I was given enough assignments during my clinical rotation.

##### Factor V: Motivation \ learning skills:

• I adequately know my learning needs.
• I know my limitations.
• I am eager to learn.
• I am able to look for new information.
• I come to the clinical sessions prepared and ready.
• I enjoy learning in clinical sessions.
• I am able to express myself and show confidence.

#### Part 2: The student patient contact:

Students instructed to report the number of their meaningful clinical patient encounters. The meaningful patient encounter is defined when the student is involved in obtaining patient interview or performing physical examination and not one where a patient described or presented in the student presence.

The student asked to answer the following questions:

• Please indicate the number of patients whose medical history you took by yourself during Junior Internal Medicine Clerkship;
• Please indicate the number of patients for whom you performed a physical examination on your own during Junior Internal Medicine Clerkship.

#### Part 3: Importance of learning methods in preparation for clerkship exams

The students asked to rank on scale 1-7 the following teaching components in order of importance in preparation for post clerkship examinations:

##### Preparation for NBME exam:

• Textbook and online resources
• Practice MCQ questions
• Faculty teaching (morning report, tutorials, etc.)
• Clinical experience (patient workups, bedside teaching, etc.)

##### Preparation for OSCE exam:

• Textbook and online resources
• Practice with other students on history taking and physical examinations
• Faculty teaching (morning report, tutorials, etc.)
• Clinical experience (patient workups, bedside teaching, etc.)
