# Supplementary material for: Does performance on United States national board of medical examiners reflect student clinical experiences in United Arab Emirates?
Source: MedEdPublish (2016). 2019 Apr 1;8:4. Originally published 2019 Jan 4. [Version 2] doi: 10.15694/mep.2019.000004.2 (PMC10712583; doi:10.15694/mep.2019.000004.2)
Supplement: Peer review history

# Reviewer report: Reviewer response for version 2

**DOI:** 10.21956/mep.20238.r31371

**Published:** 2019-06-26

**Authors:** Richard Hays

**Affiliations:** 1 James Cook University

This review has been migrated. The reviewer awarded 4 stars out of 5 The title of this paper raises the question: What does the USA national licensing exam have to do with the UAE? The answer is both not much and a lot, depending on the lens in the viewer. The exam itself is strongly based on USA medical education - teaching, learning experience and assessment. Because the exams determine eligibility to practice, a huge business has grown out of preparation for the assessments rather than (or in parallel to) local institutional assessments. US institutions 'buy' or follow closely the NBME item style model and US students often purchase 'shelf' tests for additional practice. Because so many international medical graduates want to work in the USA, many international medical programs use the retired item bank to assist their students prepare for this. Further, many medical schools around the world now use the NBME retired item bank (the questions are of high quality and come with item performance data) as formative assessment. Anecdotally, a similar mis-match between institutional and NBME assessment scores has been found in Australia. This most likely reflects differences in curriculum sequence, emphasis and assessment, rather than a comment on the quality of either Australian or USA medical graduates. The most important message here is that assessment needs to reflect the learning experiences of medical students - constructive alignment is important! This paper is therefore a welcome addition to the literature, as it should provoke thinking about what band how we assess medical students. It would be interesting to hear of similar experiences in other countries.

Reviewer Expertise:

NA

No decision status is available

# Reviewer report: Reviewer response for version 2

**DOI:** 10.21956/mep.20238.r31372

**Published:** 2019-06-14

**Authors:** Ken Masters

**Affiliations:** 1 Sultan Qaboos University

This review has been migrated. The reviewer awarded 4 stars out of 5 An interesting paper dealing with the use of the US national board of medical examiners reflect student clinical experiences in the UAE, and the suitability of the examination outside of the US.The authors begin by describing the problem of the suitability of the examination. Although there are many international standards, there are also many variations, and there is great difficulty in standardising this type of examination, even if the focus is on knowledge – if it were easy, there would simply always be only one international qualifying examination. Medical schools have to teach curricula that take into account the local environment and culture, and examinations need to match the teaching. This, in of itself, points to the type of problem facing the researchers in the UAE.The findings of a lack of correlation between the NBMESE scores and the students’ clinical experience is problematic, but the authors do not automatically assume that the fault lies with the NBMESE, as the students also reported low authenticity of the clinical experiences, and the authors note that this could have a negative impact on the scores.I think the main value of the paper is that the authors have highlighted a problem in the use of the NBMESE in the UAE. (It should serve as a heads up to other countries and regions using the NBMESE (and other international examinations) that perhaps a similar investigation should be undertaken). I would suggest that the next stage is to try to investigate much more deeply exactly where the problem lies. For this, a more detailed analysis of the various testing systems would need to be undertaken. This may require a much closer collaboration with the NBMESE to examine the test items, the question format, the language and terminology, the assumptions, etc., and how these match to what is taught. The solution may be local adjustments of the test (which is fairly common in other fields), adjustments to the teaching methods and students’ training and other examinations, or (most likely), a combination of all.Either way, a useful paper that points ahead to a great deal of work to be done. I look forward to seeing a more detailed study in the future, in which the researchers have attempted a resolution to the problem.

Reviewer Expertise:

NA

No decision status is available

# Reviewer report: Reviewer response for version 2

**DOI:** 10.21956/mep.20238.r31370

**Published:** 2019-04-01

**Authors:** Magnus Hultin

**Affiliations:** 1 Umeå University

This review has been migrated. The reviewer awarded 3 stars out of 5 Thank you for an interesting paper on the discrepancies between performances on NBMESE and the locally defined OSCE. To better understand the differences it would be helpful if you either had references to the full curriculums to both NBMESE and to the comparable curriculum at the UAB University? Are the observed differences comparable to the problems observed for IMGs taking US exams?

Reviewer Expertise:

NA

No decision status is available

# Reviewer report: Reviewer response for version 1

**DOI:** 10.21956/mep.19119.r27586

**Published:** 2019-01-10

**Authors:** Michael Ryan

**Affiliations:** 1 Virginia Commonwealth University School of Medicine

This review has been migrated. The reviewer awarded 2 stars out of 5 Summary: The authors studied the relationship between third year medical student performance on the NBME subject examination in Internal Medicine with their perception of clinical experiences at a single institution in the United Arab Emirates. The authors found that students performed markedly worse on the subject examination that the NBME-provided means. They did not find a relationship between scores on this examination with the perceived quality of clinical experiences. The authors conclude that the subject examination may not accurately represent the patient care experiences of learners in UAE and that the country should consider the development of an alternative examination for their country. Strengths: The authors provide a good review of the literature on the topic and selected an instrument with validity evidence to measure clinical experiences of their learners. In general, the statistical analysis is appropriate and well thought out.Areas for improvement: The premise of this manuscript is that the subject examination test knowledge acquired during clinical care experiences and that this is the case in the US. I wouldn't say that is accurate. The subject examinations are designed to assess knowledge obtained by the conclusion of the clerkship. Whether that is obtained in the course of caring for patients vs. self-reading is not specifically stated by the NBME. In addition to that issue, the authors could strengthen this paper by looking at other measures of knowledge in their population to see if this is an issue with the Medicine examination specifically, or all examinations intended for a US audience. The scores were so low here that it made me wonder what the scores on exams like Step 1, Step 2, and other subject exams were. We would need to know what those scores are like to determine if this is an issue with clinical experience or a more global issue with the content of the exam, familiarity, etc. Comments by sectionAbstract: Represents the manuscript relatively well. I would have liked to see the data regarding performance of this population compared to the mean though. Introduction: - I was confused by the statement that the UAEU undergraduate medical curriculum is benchmarked with the US national core medical curriculum. Similarly there's a statement that the subject exam is benchmarked to the US curriculum. There is no standardized US curriculum. Methods- Expand on the model of teaching in UAEU. The authors state “apprenticeship,” but follows by saying “shadowing.” - Several elements of this section appear to be results. For example, the study population, % men, response rate to survey, etc- Statistical analysis chosen requires more detail. - Number of patients reported relies on self-report. This seems to be a significant threat to validityResults- The authors remark on pre-clerkship scores. What are these? - The authors analyze by "high" and "low" achievers but don't discuss how these groups were created.- Performance on the subject exam is incredibly low compared to the national average. This raises the concern of whether this performance has anything to do with the experience on the medicine rotation or reflects a larger issue with test familiarity or overall knowledge base. Discussion/Conclusions- The premise that UAE should consider developing a test that better reflects learning among its students is reasonable. But, I don't know that it is supported by the findings here. The authors showed that students performed poorly on the NBME examination, but that alone doesn't mean the examination is not appropriate. It depends on the goal of assessment. If the goal is to prepare students for USMLE, there is substantial evidence that the subject examinations are correlated with that examination. If the goal is to mirror clinical experiences, then I agree, they may want to pursue a test that is more related to the clinical experience.

Reviewer Expertise:

NA

No decision status is available

# Reviewer report: Reviewer response for version 1

**DOI:** 10.21956/mep.19119.r27587

**Published:** 2019-01-06

**Authors:** P Ravi Shankar

**Affiliations:** 1 American International Medical University

This review has been migrated. The reviewer awarded 4 stars out of 5 I enjoyed reading this article examining the correlation between student performance in the NBME subject exam and student clinical experiences. NBME offers exams which are standardized and which could be administered easily using a secure platform. NBME is widely administered in Caribbean offshore medical schools. There is a tendency toward standardization and certifying exams the world over. I am of the opinion that big and diverse nations like the United States, China and India will have to be careful while designing exit exams as the clinical experience in different parts of the country and in different medical schools may be different. This article points out how NBME may not accurately reflect the clinical experiences of students in the United Arab Emirates. The authors base this assumption primarily on the CLEQ and the logged patients. The NBME measures primarily knowledge and the knowledge component could be tested using another locally created exam. Single response MCQs are easy to grade, objective and can cover a wide range of topics but may not test all knowledge domains or competencies. The authors mention the limitations of their study clearly. This article will be of interest to all medical educators.

Reviewer Expertise:

NA

No decision status is available
